# Transcatheter Closure of Patent Ductus Arteriosus under Echocardiography Guidance: A Randomized Controlled Noninferiority Trial

**DOI:** 10.1155/2020/4357017

**Published:** 2020-10-03

**Authors:** Cheng Wang, Fengwen Zhang, Wenbin Ouyang, Guangzhi Zhao, Wenxin Lu, Mengxuan Zou, Xiangbin Pan

**Affiliations:** National Center for Cardiovascular Disease, China and Fuwai Hospital, Chinese Academy of Medical Sciences and Peking Union Medical College, Beijing 100037, China

## Abstract

**Background:**

Percutaneous occlusion under fluoroscopy guidance has become the preferred method for the treatment of patent ductus arteriosus (PDA). To avoid radiation exposure and contrast agent use, PDA occlusion under transthoracic echocardiography (TTE) guidance was conducted.

**Objectives:**

We assessed the hypothesis that the success rate of percutaneous PDA occlusion under TTE was noninferior to that under fluoroscopy guidance.

**Methods:**

In this single-center trial, 100 patients were randomly assigned in a 1 : 1 ratio to the TTE group (*n* = 50) or to the fluoroscopy group (*n* = 50). The primary endpoint was the success rate of occlusion, with the noninferiority margin set at 8% for the between-group difference in intention-to-treat analysis. Secondary endpoints were hospitalization duration, cost, procedure time, and rate of adverse events including occluder migration, hemolysis, peripheral vascular complications, and residual shunt at 1-month and 12-month follow-up.

**Results:**

Patient, defect, and device characteristics were similarly distributed between groups. The success rate of occlusion was 98% for the TTE group and 100% for the fluoroscopy group (absolute difference: −2%; 95% confidence interval: −5.9% to 1.9%). Cost and procedure duration were significantly lower in the TTE group, without adverse events in either group at a median of 12.0 months (range, 10.0–15.5 months) of follow-up.

**Conclusion:**

Percutaneous PDA occlusion can be performed via TTE guidance safely and effectively, and the success rate of the TTE-guided procedure was noninferior to that under fluoroscopy guidance, with reduced cost and procedure time. The trial is registered with http://www.chictr.org.cn (ChiCTR-ICR-15006334).

## 1. Introduction

PDA is considered a form of congenital heart disease. In the past decades, transcatheter closure has become the leading approach to closure of most PDAs [1, 2]. Although the procedure currently can be performed with limited fluoroscopic exposure and nonionic contrast administration, the potential for injury remains as reflected in a recent large-scale study of adults with congenital heart disease which showed that low-dose ionizing radiation from cardiac procedures was associated with incident cancer risk [3]. Percutaneous and nonfluoroscopical (PAN) procedure for structural heart disease such as ASD, PDA, and VSD has been established successfully to reduce potential injury related to fluoroscopy and angiography. We have reported our successful experience in TTE-guided PDA occlusion previously in a retrospective way [4–6]. However, still no prospective randomized controlled study comparing TTE guidance with fluoroscopy guidance in PDA occlusion has been conducted, and controversy persists on how the success rate of transcatheter closure of PDA under TTE guidance would be compared with that under fluoroscopy guidance [7, 8].In this randomized controlled study, we assessed the hypothesis that the success rate of percutaneous PDA occlusion using TTE guidance was noninferior to that of fluoroscopy guidance.

## 2. Methods

### 2.1. Patients

This prospective, randomized controlled trial was undertaken at Fuwai Hospital, Beijing, China. Enrolment began in May 2015 and ended in May 2017. A total of 100 patients were enrolled. Criteria for inclusion were as follows: patient weight ≥8 kg and presence of clinical symptoms or echocardiographic evidence of cardiac overload such as enlarged left ventricle. Patients were excluded if they presented with infective endocarditis or severe pulmonary hypertension with right to left shunt, were PDA-dependent survivors, or required surgery to treat a heart deformity. Eligible patients underwent TTE examination to measure the PDA length and narrowest diameter.

The study was reviewed by the China-registered clinical trial ethics review committee. All patients provided written informed consent. An independent clinical events committee reviewed and adjudicated all adverse events. An independent data safety monitoring board met regularly to review study data and to recommend any changes to the protocol.

### 2.2. Randomisation and Allocation Concealment

After baseline screening, patients were randomly assigned by a computer-generated randomisation sequence to TTE or fluoroscopy groups in a 1 : 1 ratio. An independent statistician who had no involvement in the design or analysis of the study generated the randomisation sequence. The patients' case numbers and date of birth were entered, and then, the patient was allocated to TTE or fluoroscopy groups. Participants and clinicians were not blinded to treatment assignment.

### 2.3. Study Endpoints and Definitions

The primary endpoint was procedural success, defined as successful implantation of the device under the guidance of TTE or fluoroscopy, without device migration. The secondary endpoints were hospitalization duration, cost, procedure time, and rate of adverse events including occluder migration, hemolysis, peripheral vascular complications, and residual shunt at 1-month and 12-month follow-up postoperatively.

### 2.4. Procedures

**Figure 1:**
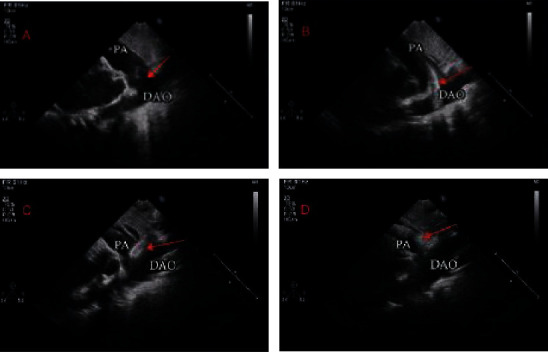
Transcatheter closure of a PDA under TTE guidance via femoral vein. (a) The PDA (arrow) was measured using the parasternal short-axis view. (b) The parasternal short-axis view showed that the guide wire (arrow) was located within the descending aorta. (c) Release of the aortal side disk of the occluder (arrow). (d) Release of the pulmonary side disk of the occluder (arrow). DAO = descending aorta; PA = pulmonary artery.

Procedures for TTE guidance have been published previously [5]. The intervention procedures took place in the ordinary operation room. All patients underwent preoperative and intraoperative TTE to measure the PDA length and narrowest diameter (Figure 1(a)). With the patient in a supine position, heparin (80 IU/kg) was administered and the right femoral vein was punctured under local anesthesia (cooperative patients) or sedation using propofol with spontaneously breathing (uncooperative patients) to insert a 6 F arterial sheath. A 6 F right coronary artery JR 3.5 catheter and a guide wire were delivered through the arterial sheath (Figure 1(b)). Under TTE guidance, the catheter and wire were advanced into the right ventricle via the tricuspid valve and then into the main pulmonary artery through the pulmonary valve. After withdrawing the wire, a complete hemodynamic assessment including shunt calculation, pressure measurements, and pulmonary vascular resistance calculation was made. Then, the catheter was exchanged for a 6 F multipurpose catheter. Under guidance of TTE using a long-axis section at the level of great vessels, the guidewire was advanced into the PDA and placed in the descending aorta. Then, this catheter was exchanged for the delivery sheath over the guidewire, leaving the sheath in the descending aorta. The proper-size occlusion device (4 mm larger than the narrowest size of the PDA, PDA occluder, Shape Memory Alloy Co., Ltd, Shanghai) was chosen and pulled into the loader. The loader was introduced into the delivery sheath, and the device was advanced into the descending aorta. The sheath was retracted until the retention disk was opened in the proximal descending aorta (Figure 1(c)). The sheath with the delivery cable in it was pulled back until the retention disk was snug against the aortic end of the ampulla. While maintaining tension on the delivery cable, the delivery sheath was retracted into the pulmonary artery to release the tubular frame of the occluder into the PDA (Figure 1(d)). Once the proper device position was confirmed by TTE without significant residual shunt or flow acceleration, the device was released by turning the cable counterclockwise. The cutaneous wound was bandaged after hemostasis was achieved.

In the fluoroscopy group, patients underwent the routine catheterization protocol for PDA occlusion. To summarize, a right heart catheterization was performed via the femoral vein. A biplane descending aortogram was performed in order to profile the ductus via the femoral artery. Next, a multipurpose catheter and wire were passed through the ductus to the descending aorta from the venous side, which was exchanged for the delivery sheath. Then, the proper-size device (4 mm larger than the narrowest size of the PDA) was pulled into the loader and was advanced into the descending aorta through the delivery sheath. After release of the device, a repeated angiogram was then performed to confirm appropriate positioning of the device and to assess the degree of residual shunt.

### 2.5. Statistical Analysis

An intention-to-treat analysis was performed for the prespecified comparisons of the success rate of transcatheter closure with TTE guidance versus fluoroscopy guidance. The prospective randomized controlled trial was designed to test the hypothesis that TTE guidance was noninferior to fluoroscopy guidance for procedural success. Study analyses were conducted according to a prespecified statistical plan.

Based on clinical experience, the success rate of the fluoroscopy group was expected to be 98%. A clinically recognized noninferiority margin of 8% was chosen. The initially planned sample size was 100 patients, which, at a two-sided 5% significance level, would provide more than 80% power to detect a difference in the procedural success rate for the comparison of TTE guidance with fluoroscopy guidance.

Continuous data are presented as mean ± standard deviation (SD) and were compared using Student's *t*-test after testing for normal variance or a nonparametric test. Categorical data are presented as number and percentage. A two-tailed *P* < 0.05 was considered statistically significant. Statistical analysis was conducted using SPSS 22.0 software.

## 3. Results

**Figure 2:**
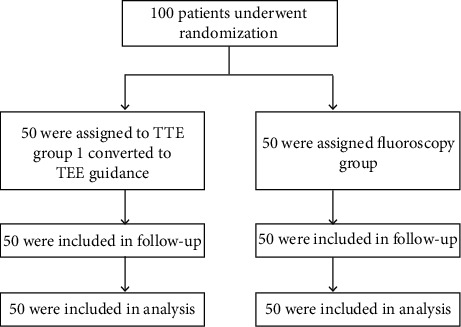
Trial profile.

The 100 eligible patients were randomized to either the TTE group (*n* = 50) or the fluoroscopy group (*n* = 50) and analyzed on an intention-to-treat basis. Baseline characteristics were similarly distributed between the two groups (Table 1).  Figure 2 shows the trial profile.

### 3.1. Study Primary Endpoint

In the TTE group, 1 patient was switched to a transesophageal echocardiography- (TEE-) guided procedure due to poor acoustic window (patient weight of 70 kg, with a height of 155 cm) under sedation without intubation. All patients in the fluoroscopy group successfully underwent transcatheter closure.

The success rate of occlusion was 98% for the TTE group and 100% for the fluoroscopy group (absolute difference: −2%; 95% confidence interval: −5.9% to 1.9%). The upper limit of the confidence interval of 1.9% did not exceed the 8% clinically recognized noninferiority margin. Therefore, the success rates of the TTE group were noninferior to those of the fluoroscopy group.

### 3.2. Secondary Study Endpoints

Compared with the fluoroscopy group, hospitalization cost was significantly lower in the TTE group (¥27401.3 ± 4652.1 vs ¥29786.4 ± 5386.9, *P* < 0.05) and procedure time was significantly shorter (21.8 ± 6.1 min vs 32.4 ± 8.2 min, *P* < 0.01). In our hospital, the depreciation expenses for DSA (digital subtraction angiography) equipment per surgery was 1088 yuan, while it was 50 yuan in the case of ultrasonic machine. There was no significant difference in hospitalization duration (1.9 ± 0.8 day vs 1.7 ± 0.8 day, *P*=0.23; Table 2).

From the end of the procedure to discharge, there were no adverse events such as death, cardiac perforation, pericardial tamponade, tricuspid regurgitation, occluder dislodgement or deformation, hemolysis, thrombosis, or infective endocarditis in either group. In 8 cases (4 in the TTE group and 3 in the fluoroscopy group), there were traces of residual shunt immediately after the procedure, which resolved after 24 hours. Two cases in the fluoroscopy group developed hematoma in the femoral artery puncture site which resolved at discharge with a delayed hospitalization.

All the patients completed follow-up at a median of 12.0 months (range, 10.0–15.5 months) without serious adverse events such as occluder migration, hemolysis, peripheral vascular complications, and residual shunt in the two groups.

## 4. Discussion

Radiation exposure and contrast agent use can hardly be avoided in the usual way of cardiac interventional therapy, which brings potential harm to both patients and operators, especially in patients with renal dysfunction or allergic constitution [3, 9, 10]. Under echo guidance only, the PAN procedure for structural heart disease such as ASD, PDA, and VSD has been established successfully to reduce potential injury related to fluoroscopy and angiography [4–6, 11]. Here, we designed and conducted this noninferiority randomized controlled trial to further confirm the procedural success rate of PAN procedure in PDA treatment.

The results of this randomized trial indicate that the success rate of percutaneous PDA occlusion using TTE guidance was noninferior to that of fluoroscopy guidance, while being associated with reduced cost and shorter procedure time.

In this study, the success rate of percutaneous PDA occlusion using TTE guidance, which was 98%, was noninferior to that of fluoroscopy guidance (at 100%). The occluder was successfully implanted in all the patients except one in the TTE group. This patient was switched to a TEE-guided procedure due to poor acoustic window (patient weight of 70 kg, with a height of 155 cm), and the occluder was successfully implanted thereafter. The results of this study were comparable to those of other studies, and the result for the TTE group was satisfactory as the noninferiority is being accepted [12].

Three findings further favor TTE guidance. First, our data show that PDA occlusion under TTE guidance significantly reduced total cost compared with fluoroscopy guidance. Because hospital stay did not differ significantly between the two groups, savings are due primarily to tools and equipment depreciation expenses and contrast agent costs. At our hospital, the depreciation expenses for digital subtraction angiography equipment per surgery were 1088 yuan, in contrast to 50 yuan for the ultrasound machine. Obviously, the extra use of the contrast agent adds to the total cost of fluoroscopy-guided procedure. Second, the procedural time for occlusion under TTE guidance was shorter than that under fluoroscopy guidance. In our center, the PAN procedure for structural heart disease such as ASD, PDA, and VSD has been successfully and regularly applied and all the TTE-guided procedures were carried out by experienced PAN procedure operators, which contributed a lot to the satisfying procedural time for the TTE guidance group. Besides, for TTE-guided occlusion, there was only one puncture site, i.e., femoral vein. For fluoroscopy-guided occlusion, usually both femoral vein and artery were punctured to provide aortogram while operating. Third, two patients in the fluoroscopy guidance group developed hematoma at the femoral artery puncture site. The femoral artery was left untouched in the TTE guidance group, and no hematoma was seen at the femoral vein puncture site.

The most frequent complications associated with percutaneous closure of PDA include device embolization, protrusion of the retention disk of the device into the aorta producing aortic obstruction, or obstruction of a branch pulmonary artery by the device [13]. In the present study, as discussed above, no patients experienced these complications during follow-up.

This study had several limitations. Although the noninferiority in the procedural success rate of TTE guidance compared with fluoroscopy guidance was proved, the 95% CI was wide, ranging from −5.9% to 1.9%, owing to the low number of clinical events and small sample size in this trial. Moreover, although the endpoint events were judged by an independent blinded committee, the patients and their attending physicians were not blinded for randomisation. This may have to some extent influenced the timing of hospital discharge, procedural time, and total cost. Finally, the study is also limited by its single-center design with an ethnically homogeneous population and all the operations for the TTE group were done by operators experienced in the PAN procedure. Larger multicenter studies with longer-term follow-up are warranted.

## 5. Conclusion

TTE-guided PDA occlusion appeared safe and yields a procedural success rate comparable to that with fluoroscopy-guided operation. In addition, omitting angiography and femoral artery puncture lead to reduced cost and shorter procedure time. Percutaneous PDA occlusion using TTE guidance might provide an alternative strategy to traditional fluoroscopy guidance while avoiding radiation exposure and contrast agent use.

## Figures and Tables

**Table 1:** General patient, defect, and device characteristics.

	TTE group (*n* = 50)	Fluoroscopy group (*n* = 50)	*P* value
Sex (male, %)	12, 24%	15, 30%	0.499
Age (year)	10.5 ± 12.7 (1–51)	15.2 ± 16.4 (1–66)	0.115
Weight (kg)	29.6 ± 19.9 (8.0–70.0)	32.5 ± 23.1 (8.0–108.0)	0.572
PDA size (mm)	4.8 ± 1.3 (3–9)	4.7 ± 2.2 (2–9)	0.156
Occluder size (mm)	10.5 ± 1.4 (8–14)	10.2 ± 2.3 (8–16)	0.153

**Table 2:** Comparison of secondary endpoints between two groups.

	TTE group (*n* = 50)	Fluoroscopy group (*n* = 50)	*P* value
Procedure time (min)	21.8 ± 6.1 (10–35)	32.4 ± 8.2 (17–50)	<0.01
Hospitalization duration (day)	1.9 ± 0.8 (1∼4)	1.7 ± 0.8 (1～4)	0.23
Hospitalization cost (¥)	27401.3 ± 4652.1 (10640.0–37128.4)	29786.4 ± 5386.9 (18709.1–40900.6)	0.02

## Data Availability

The data used to support the findings of this study are available from the corresponding author upon request.

## Abbreviations

PDA: Patent ductus arteriosus
TTE: Transthoracic echocardiography
ASD: Atrial septal defect
VSD: Ventricular septal defect
SD: Standard deviation
TEE: Transesophageal echocardiography
PAN: Percutaneous and nonfluoroscopical.

## Supplementary Materials

Supplementary Materials Supplemental video: transcatheter closure of a PDA under TTE guidance via the femoral vein.
